# Distress tolerance and emotional regulation in individuals with alcohol use disorder

**DOI:** 10.3389/fpsyt.2023.1175664

**Published:** 2023-05-12

**Authors:** Justyna Zaorska, Małgorzata Rydzewska, Maciej Kopera, Paweł Wiśniewski, Elisa M. Trucco, Paweł Kobyliński, Andrzej Jakubczyk

**Affiliations:** ^1^Department of Psychiatry, Medical University of Warsaw, Warsaw, Poland; ^2^Department of Psychology, Center for Children and Families, Florida International University, Miami, FL, United States; ^3^Department of Psychiatry, Addiction Center, University of Michigan, Ann Arbor, MI, United States; ^4^Laboratory of Interactive Technologies, National Information Processing Institute, Warsaw, Poland

**Keywords:** distress tolerance, emotional dysregulation, alexithymia, depressive symptoms, alcohol use disorder

## Abstract

**Background:**

Previous research suggests that low distress tolerance may be associated with poor emotion regulation, contribute to drinking to cope motives, and predict alcohol-related problems in non-clinical populations. However, little is known about the ability to tolerate distress among individuals with alcohol use disorder (AUD) and its association with emotional dysregulation. The aim of this study was to examine the link between emotional dysregulation and a behavioral measure of distress tolerance among individuals with AUD.

**Methods:**

The sample consisted of 227 individuals with AUD enrolled in an 8-week abstinence-based inpatient treatment program. Behavioral distress tolerance was assessed using a test of ischemic pain tolerance and the Difficulties in Emotion Regulation Scale (DERS) was used to assess emotion dysregulation.

**Results:**

Distress tolerance was significantly associated with emotional dysregulation even when accounting for alexithymia, depressive symptomatology, age, and biological sex.

**Conclusion:**

The current study provides preliminary support for an association between low distress tolerance and emotion dysregulation in a clinical group of patients with AUD.

## Introduction

The multidimensional nature of distress tolerance is emphasized in the current literature. Thus, formulating a single overarching theory of distress tolerance is challenging. For example, Simons and Gaher (1) conceptualize distress tolerance as an individual’s ability to tolerate aversive states. In their view, distress tolerance reflects the ability to tolerate and accept negative emotions, emotion regulation strategies, and the degree to which attention is absorbed by negative emotions and its interference with functioning. In previous studies, distress tolerance is believed to reflect two components: (1) the perceived competence to withstand an aversive state (e.g., negative emotions, physical discomfort) and (2) the behavioral act itself of withstanding aversive states. It follows that the methodology used to measure these two components of distress tolerance differ; perceived distress tolerance is commonly measured through self-report questionnaires, while behavioral distress tolerance is assessed via tasks exposing participants to aversive stimuli, such as pain (2).

A sizable portion of studies show that an important clinical correlate of distress tolerance is problematic alcohol drinking. Findings demonstrated that distress tolerance may predict heavy episodic drinking among college students, although Padrelli and colleagues observed this association only among females (3). Low distress tolerance may contribute to drinking to cope motives, as well as predict negative alcohol drinking consequences (such as blacking out, driving while intoxicated, risky sexual activity) and alcohol-related problems in non-clinical populations (4–6). Moreover, depressive symptoms in individuals with low (but not high) distress tolerance were associated with problematic alcohol use in a healthy community sample (7). Holzhauer and colleagues (8) observed that in a non-clinical population of women, low subjective distress tolerance was associated with more alcohol-related consequences. However, there is a limited number of studies examining distress tolerance in individuals with an alcohol use disorder (AUD). A notable exception is a single randomized controlled trial demonstrating a negative association between self-reported distress tolerance and substance use craving among individuals with a substance use disorder (9).

A suggested explanation of the association between lower distress tolerance and alcohol drinking is that lower levels of distress tolerance may lead to poorer emotional regulation, which in turn increases the risk of excessive alcohol drinking. Gratz and Roemer (10) define emotion regulation as awareness, acceptance and understanding of emotions, the ability to control impulsive behaviors while experiencing distress, and the use of adaptive strategies to modulate emotional responses. Importantly, emotion dysregulation is a well-recognized and important risk factor in the development of AUD, as well as an indicator of poor treatment outcomes (11, 12). The association between low distress tolerance and poor emotion regulation was demonstrated in the context of alcohol drinking in non-clinical samples. For example, a study conducted among men with problematic alcohol use found that distress intolerance impacted the association between negative affect and craving (13). In a study by Howell and colleagues (6) distress tolerance was related to coping motives for alcohol use in a community sample of young adults. In another study (14) conducted among psychology undergraduates, distress tolerance mediated the association between severity of depressive symptoms and alcohol-related problems. In a sample of individuals exposed to at least one traumatic life event, distress tolerance partially mediated the association between impulsivity and alcohol use coping motives (15). In this study, higher levels of impulsivity were significantly associated with lower distress tolerance. In general, there is a convincing amount of evidence that lower distress tolerance is associated with clinical manifestations of low behavioral control. A recent meta-analysis showed that low distress tolerance is a common, transdiagnostic feature of problematic substance use, disordered eating behaviors, and borderline personality disorder, which are all characterized by high emotional dysregulation and poor behavioral control (16).

Accordingly, it is plausible that low levels of distress tolerance would be associated with higher levels of emotional dysregulation among individuals with AUD. Surprisingly, to the best of our knowledge, no studies have directly empirically tested this association. The aim of the current study was to examine associations between emotional dysregulation and a behavioral measure of distress tolerance among individuals with AUD. We hypothesized that among individuals with AUD lower distress tolerance will be associated with worse emotion regulation.

## Materials and methods

The study received approval from the Bioethics Committee at the Medical University of Warsaw. For this study, a group of 227 individuals aged 43.85 ± 11.0 with severe AUD entering an abstinence-based, drug-free, inpatient treatment program in Warsaw, Poland, was recruited. The average years of education for participants was 12.0 ± 3.9, which corresponds to complete secondary education in Poland. The AUD diagnosis was obtained through the International Classification of Diseases and Related Health Problems 10th Revision (17) upon treatment admission, and was later confirmed through the MINI International Neuropsychiatric Interview (18). Exclusion criteria included the following: a clinically significant cognitive deficit (<25 on the Mini-Mental State Examination) (19), a history of psychosis, co-occurring current psychiatric disorders requiring medication, current use of analgesics, and co-occurrence of substance use/dependence other than nicotine. In terms of drinking characteristics, participants reported: (1) first experiencing drinking problems during early adulthood (25.7 ± 9.6 years of age); (2) the duration of alcohol abstinence prior to completing the study procedures was 49.2 ± 45.1 days. Study procedures were performed during the first two weeks after treatment admission. Given the overrepresentation of men in substance use treatment programs in Poland, a large portion of the sample comprised White men (85.2%).

### Measures

In contrast to self-reported measures of distress tolerance, which assess *perceived* ability to withstand aversive states, behavioral measures assess true ability to withstand aversive states (such as pain or frustration) while being exposed to them. In this study, behavioral distress tolerance was assessed using a test of ischemic pain tolerance (IPT). In this procedure, the nondominant arm of the participant is elevated above the heart for 30 s to allow for the blood to drain, after which a blood pressure cuff is positioned and inflated to 200 mm Hg. Participants then perform hand grip exercises for a specified duration and effort level and are instructed to continue until the pain becomes intolerable, but not in excess of 5 min. Distress tolerance was then operationally defined as the number of seconds to pain tolerance after beginning the handgrip exercise. Physical pain is inseparably linked to concurrent negative emotional states (20). Therefore, while some research utilizes measures of pain tolerance only for the purpose of pain assessment, in broader, holistic conceptualizations, pain tolerance is considered an established measure of behavioral distress tolerance (2). Importantly, evaluating pain as a measure of distress tolerance may be especially appropriate in this sample given the high prevalence of physical pain in individuals with AUD and its negative influence on the course of the disorder [see for example (21)].

The Polish version of the Difficulties in Emotion Regulation Scale (DERS) (10, 22) was used to assess emotion dysregulation. For the purpose of the current study, a total DERS score was used (Cronbach’s alpha = 0.92). Higher scores on the DERS indicates worse emotion regulation.

The total score of the Polish version of the Toronto Alexithymia Scale [TAS-20; (23, 24)] was utilized as a self-report measure of alexithymia (Cronbach’s alpha = 0.82). Depressive symptom severity was evaluated with the Polish version of the Beck Depression Inventory [Cronbach’s alpha = 0,93; (25)]. The analyses covaried for the duration of alcohol abstinence prior to completing the study procedures, alexithymia and depressive symptoms because they have been strongly linked to symptoms of AUD, pain tolerance and emotion dysregulation (20, 26–29).

### Statistical analyses

The analyses focused on examining the association between emotional dysregulation (DERS) and distress tolerance among individuals with AUD. First, correlations between emotional dysregulation, distress tolerance, depressive symptoms (BDI), and alexithymia (TAS), were examined to evaluate the rationale for including them in the modeling process. Next, a three-iteration hierarchical stepwise linear regression with forward selection was conducted to assess the association between emotional regulation in individuals with AUD (dependent variable) and distress tolerance, while adjusting for alexithymia (TAS) and severity of depressive symptoms (BDI). Initially, demographic variables (age and biological sex) as well as length of abstinence prior to the study procedures were included in the model, but they were ultimately removed due to their unrelatedness to the variable of interest, as indicated by preliminary analyses.

## Results

The average score in the pain ischemic task was 70.2 ± 26.2 s. The average BDI score was 17.9 ± 10.1, the average DERS total score was 85.7 ± 20.3, and the average TAS score was 57.2 ± 11.7.

A significant negative correlation emerged between emotional dysregulation and distress tolerance (*r* = − 0.199; *p* = 0.004). In addition, a significant positive correlation was found between emotional dysregulation (higher DERS total score) and depressive symptomatology (BDI, *r* = 0.381; *p* < 0.001) and alexithymia (TAS; *r* = 0.468; *p* < 0.001). Correlations are presented in Table 1.

**Table 1:** Correlation matrix for the associations between emotional dysregulation, depressive symptoms, alexithymia, and distress tolerance in individuals with alcohol use disorder.

	Emotional dysregulation	Depressive symptoms	Alexithymia	Distress tolerance
Emotional dysregulation	1	***r* = 0.381** ***p* < 0.001**	***r* = 0.468** ***p* < 0.001**	***r* = − 0.199** ***p* = 0.004**
Depressive symptoms	***r* = 0.381** ***p* < 0.001**	1	***r* = 0.346** ***p* < 0.001**	*r* = − 0.094 *p* = 0.178
Alexithymia	***r* = 0.468** ***p* < 0.001**	***r* = 0.346** ***p* < 0.001**	1	*r* = − 0.051 *p* = 0.469
Distress tolerance	***r* = − 0.199** ***p* = 0.004**	*r* = −0.094 *p* = 0.178	*r* = − 0.051 *p* = 0.469	1

The bolded texts refer to *p* values lower than 0.05.

Distress tolerance was significantly associated with emotional dysregulation when accounting for alexithymia and depressive symptomatology (for details see Table 2). The final linear regression model, obtained after the third iteration of the forward selection procedure, explained 33.9% of the variance in emotional dysregulation (*R*^2^ = 0.339; *F*[3,198] = 33.786; *p* < 0.001). Distress tolerance accounted for 2.6% of the variance in the dependent variable (Δ*R*^2^ = 0.026; *F*[1,198] = 7.798; *p* = 0.006).

**Table 2:** Hierarchical stepwise linear regression assessing the association between emotional regulation and distress tolerance.

Model iteration	ΔR^2^	*F*	*p*	Independent variable	B (unstand. Beta)	SE B	95% CI B	*t*	*p*	VIF
1	0.247	65.511	<0.001	Alexithymia (TAS)	0.862	0.107	0.652 1.072	8.094	<0.001	1.000
2	0.066	19.046	<0.001	Alexithymia (TAS)	0.691	0.109	0.475 0.906	6.319	<0.001	1.148
				Severity of depressive symptoms (BDI)	0.557	0.128	0.305 0.808	4.364	<0.001	1.148
**3**	**0.026**	**7.798**	**0.006**	**Alexithymia (TAS)**	**0.685**	**0.107**	**0.473 0.897**	**6.376**	**<0.001**	**1.149**
				**Severity of depressive symptoms (BDI)**	**0.526**	**0.126**	**0.278 0.775**	**4.179**	**<0.001**	**1.157**
				**Distress tolerance**	**−0.127**	**0.045**	**−0.217–0.037**	**−2.793**	**0.006**	**1.010**

BDI, Beck Depression Inventory; TAS, Toronto Alexithymia Scale. The bolded texts refer to *p* values lower than 0.05.

## Discussion

In this study the association between distress tolerance and emotion dysregulation was assessed adjusting for alexithymia and depressive symptoms within individuals with AUD. A significant negative correlation was detected between emotional dysregulation and distress tolerance and a significant positive correlation emerged between depressive symptomatology and alexithymia. Furthermore, the multivariate models revealed that distress tolerance was significantly associated with emotional dysregulation even when accounting for alexithymia and depressive symptomatology. To the best of our knowledge, this is the first study to investigate associations between behavioral distress tolerance and emotion dysregulation in a clinical group of patients with AUD.

As mentioned previously, the association between distress tolerance and emotion dysregulation has been established both in clinical and non-clinical samples. Studies suggest that individuals with lower distress tolerance more commonly use maladaptive strategies of emotion regulation (e.g., suppression, avoidance, rumination) (30) and are more likely to engage in impulsive, non-planned behaviors (15). Consistently, prior work demonstrated that distress tolerance may predict heavy drinking and influence alcohol related outcomes (e.g., engage in risky drinking behaviors) as alcohol drinking may be perceived both as a maladaptive way of regulating emotions, as well as a manifestation of behavioral dyscontrol (high impulsivity). Additionally, poor emotion regulation, as well as distress intolerance has been associated with drinking to cope with negative emotions. Khan and colleagues (5) suggested that distress tolerance may contribute to alcohol use related problems via drinking to cope strategies. However, to date all studies concerning distress tolerance, emotion regulation, and alcohol drinking have focused on social drinkers and individuals with problematic alcohol drinking out of those with an AUD diagnosis. Plausibly, low distress tolerance in individuals with AUD may be of special, higher clinical significance compared to individuals without AUD, as AUD is commonly accompanied by elevations in psychological (negative affect) and somatic (hyperalgesia) distress, which are components of alcohol withdrawal – the phenomenon which is not present among individuals who engage in problematic drinking without a clinical diagnosis.

In addition, prior work has focused largely on self-report measures of distress tolerance, which may not necessarily reflect the true ability to tolerate distress, as prior work has found discrepancies between subjective and more objective measures. Therefore, our research adds to current knowledge by providing evidence for an association between distress tolerance and emotion dysregulation within a clinical group of individuals with AUD. Moreover, in contrast to previous studies which utilized mainly self-report measures (6), the current study assessed distress tolerance using a behavioral tool when exposed to pain, which is a real-life somatic and emotional stressor commonly reported by individuals with AUD (21, 31). Also, compared to survey measures that likely reflect perceptions of general distress tolerance, behavioral measures of distress tolerance may be more reflective of real-life circumstances as distress is a state-related (i.e., temporary) experience.

Although this is a preliminary study, several clinical implications can be gleaned from findings in the context of prior work. For example, lower behavioral distress prior to receiving a cognitive-behavioral therapy-based treatment was associated with greater substance use among individuals with PTSD (32). This finding underlines how the ability to tolerate distress can impact treatment outcomes, including substance use. It follows that addressing distress tolerance among individuals enrolled in AUD therapeutic programs may have utility; yet, more research in this area is needed.

There are several study limitations that are worth noting. First, we did not evaluate symptom validity. Moreover, distress tolerance accounted only for 2.6% of the variance in the dependent variable (Δ*R*^2^ = 0.026; *F*[1,198] = 7.798; *p* = 0.006). However, the change in R square value was significant, which supports the presence of the postulated association between emotion regulation and distress tolerance. Still, there are several different variables that may contribute to emotion dysregulation in sample of individuals with AUD together with distress tolerance which we did not assess. For example, drinking to cope motives or coexisting pain conditions (33). On the other hand, acute withdrawal symptoms (associated with hyperalgesia) and pain conditions requiring analgesic treatment were an exclusion criteria in our study, which should minimize the impact of pain conditions in our sample. Our sample was comprised of individuals with a severe course of AUD, admitted to an inpatient, abstinence-based program. Since individuals with AUD represent a heterogenous group, results may not generalize to individuals with a milder course of AUD. In addition, our sample constitutes a specific group of individuals who sought treatment. Plausibly, this is a group of patients suffering severe consequences of drinking. As such, this sample endorsed very high levels of impulsivity, poor emotion regulation and distress tolerance at the beginning of the treatment. On the other hand, the fact that individuals sought treatment in and of itself indicates that individuals with an AUD express emotional awareness at least to some degree. These factors limit the generalizability of our finding for larger, non-treatment-based community samples. Moreover, the sample was primarily male and White. Thus, the results may not generalize to a more diverse sample.

In conclusion, the current study provides preliminary support for an association between low distress tolerance and emotion dysregulation in a clinical group of individuals with AUD. Work delineating possible mechanisms linking emotion dysregulation and distress tolerance among individuals with an AUD represents a fruitful direction for future investigations.

## Data availability statement

The raw data supporting the conclusions of this article will be made available by the authors, without undue reservation.

## Ethics statement

The studies involving human participants were reviewed and approved by Ethics Committee Medical University of Warsaw. The patients/participants provided their written informed consent to participate in this study.

## Author contributions

JZ, MR, MK, PW, and AJ contributed to the acquisition of data. MK, ET, AJ, and PK provided analysis and interpretation of data. JZ and AJ managed the literature research and wrote the first draft of manuscript. ET, MR, MK, PW, and PK revised the manuscript and provided substantial input. All authors contributed to the conception, design of the work, and approved content of final version of the manuscript. All authors agreed to be accountable for all aspects of the work in ensuring that questions related to the accuracy or integrity of any part of the work are appropriately investigated and resolved.

## Funding

This study was supported by the National Science Centre grant (2017/25/B/HS6/00362; PI: Jakubczyk) and the National Institute on Minority Health and Health Disparities (U54 MD012393; Sub-Project ID:5378; Co-PIs Trucco and Matthew Sutherland).

## Conflict of interest

The authors declare that the research was conducted in the absence of any commercial or financial relationships that could be construed as a potential conflict of interest.

## Publisher’s note

All claims expressed in this article are solely those of the authors and do not necessarily represent those of their affiliated organizations, or those of the publisher, the editors and the reviewers. Any product that may be evaluated in this article, or claim that may be made by its manufacturer, is not guaranteed or endorsed by the publisher.
